# CFTR Expression Analysis for Subtyping of Human Pancreatic Cancer Organoids

**DOI:** 10.1155/2019/1024614

**Published:** 2019-05-02

**Authors:** Alexander Hennig, Laura Wolf, Beatrix Jahnke, Heike Polster, Therese Seidlitz, Kristin Werner, Daniela E. Aust, Jochen Hampe, Marius Distler, Jürgen Weitz, Daniel E. Stange, Thilo Welsch

**Affiliations:** ^1^Department of Visceral, Thoracic and Vascular Surgery, Medical Faculty and University Hospital Carl Gustav Carus, Technische Universität Dresden, Dresden, Germany; ^2^National Center for Tumor Diseases (NCT), Dresden, German Cancer Research Center (DKFZ), Heidelberg, Faculty of Medicine and University Hospital Carl Gustav Carus, Technische Universität Dresden, Dresden, Helmholtz-Zentrum Dresden-Rossendorf (HZDR), Dresden, Germany; ^3^Institute of Pathology and Tumour- and Normal Tissue Bank of the University Cancer Center (UCC), University Hospital Carl Gustav Carus, Medical Faculty, Technische Universität Dresden, Dresden, Germany; ^4^Medical Department I, Medical Faculty and University Hospital Carl Gustav Carus, Technische Universität Dresden, Dresden, Germany

## Abstract

**Background:**

Organoid cultures of human pancreatic ductal adenocarcinoma (PDAC) have become a promising tool for tumor subtyping and individualized chemosensitivity testing. PDACs have recently been grouped into different molecular subtypes with clinical impact based on cytokeratin-81 (KRT81) and hepatocyte nuclear factor 1A (HNF1A). However, a suitable antibody for HNF1A is currently unavailable. The present study is aimed at establishing subtyping in PDAC organoids using an alternative marker.

**Methods:**

A PDAC organoid biobank was generated from human primary tumor samples containing 22 lines. Immunofluorescence staining was established and done for 10 organoid lines for cystic fibrosis transmembrane conductance regulator (CFTR) and KRT81. Quantitative real-time PCR (qPCR) was performed for CFTR and HNF1A. A chemotherapeutic drug response analysis was done using gemcitabine, 5-FU, oxaliplatin, and irinotecan.

**Results:**

A biobank of patient-derived PDAC organoids was established. The efficiency was 71% (22/31) with 68% for surgical resections and 83% for fine needle aspirations. Organoids could be categorized into the established quasimesenchymal, exocrine-like, and classical subtypes based on KRT81 and CFTR immunoreactivity. CFTR protein expression was confirmed on the transcript level. CFTR and HNF1A transcript expression levels positively correlated (*n* = 10; *r* = 0.927; *p* = 0.001). PDAC subtypes of the primary tumors and the corresponding organoid lines were identical for most of the cases analyzed (6/7). Treatment with chemotherapeutic drugs revealed tendencies but no significant differences regarding drug responses.

**Conclusions:**

Human PDAC organoids can be classified into known subtypes based on KRT81 and CFTR immunoreactivity. CFTR and HNF1A mRNA levels correlated well. Furthermore, subtype-specific immunoreactivity matched well between PDAC organoids and the respective primary tumor tissue. Subtyping of human PDACs using CFTR might constitute an alternative to HNF1A and should be further investigated.

## 1. Introduction

Despite advances with multimodal treatment modalities such as the FOLFIRINOX regime, pancreatic ductal adenocarcinoma (PDAC) still remains the cancer with the worst prognosis. Today, combination chemotherapy in the neoadjuvant or adjuvant setting is critical for optimal outcome (Conroy et al. [1] and Hackert et al. [2]). However, PDAC is heterogeneous regarding its genetic alterations and molecular expression profile leading to subtype-specific responses towards single chemotherapeutic agents and survival [3–6]. Recently, an immunohistochemical (IHC) subtyping of PDAC using cytokeratin-81 (KRT81) and hepatocyte nuclear factor 1A (HNF1A) was found to match with the transcriptional subtypes quasimesenchymal (QM; KRT81^+^HNF1A^−^), classical (KRT81^−^HNF1A^−^), and exocrine-like (KRT81^−^HNF1A^+^) [4, 5]. In cohorts of surgically treated PDAC patients, the HNF1A^+^ subtype was associated with the best, whereas the KRT81^+^ subtypes with the worst survival prognosis [4, 5, 7]. Thus, early subtyping after diagnosis based on KRT81/HNF1A IHCs of PDAC could guide individualized combination chemotherapy.

Within recent years, modern patient-derived 3D cell cultures named organoids have emerged as a promising model for personalized tumor analysis and drug screening [8–11]. Organoid cultures enable tumor cultivation, propagation, and timely chemosensitivity testing [12]. Furthermore, due to the possibility of repeated freeze-thaw cycles, they allow the establishment of living tumor biobanks for large-scale testing of drug panels [13]. Based on the response evaluation, personalized treatment strategies for individual patients can be designed [14]. To date, immunosubtyping of human PDAC organoids using KRT81 and HNF1A has not been established. The immunosubtyping has further become difficult because the originally described HNF1A antibody (H-205; sc-8986) is no longer commercially available and alternative HNF1A antibodies failed to produce reproducible results. Another potential marker of the exocrine-like (HNF1A^+^) subtype—which might permit subtype differentiation—is the cystic fibrosis transmembrane conductance regulator (CFTR) [4]. However, CFTR expression in PDAC organoids and its correlation with HNF1A expression is unknown. In the normal pancreas, the cAMP-regulated chloride channel is apically expressed by the ductal epithelial cells [15]. CFTR mutations are linked to an increased risk for the development of PDAC [16].

The present study is aimed at analyzing KRT81, HNF1A, and CFTR expression in human PDAC organoids in order to enable routine organoid subtyping for personalized treatment.

## 2. Materials and Methods

### 2.1. Human PDAC Samples

This study was performed with human specimens obtained from patients admitted to the Department of Visceral, Thoracic and Vascular Surgery or the Medical Department I at the University Hospital Carl Gustav Carus, Technische Universität Dresden, Germany. All samples were diagnosed as PDAC according to the World Health Organization criteria by a board certified pathologist. Tissue collection, organoid culture, and analysis were permitted by the local ethics committee (#EK451122014 and #EK68022018).

### 2.2. Generation and Cultivation of Human PDAC Organoids

Tumor specimens were cut into pieces smaller than 1 mm^3^ and digested with dispase II (2.5 mg/ml, Roche) and collagenase II (0.625 mg/ml, Sigma-Aldrich) in DMEM/F12+++ medium (DMEM/F12 (Invitrogen) supplemented with 1x HEPES (Invitrogen), 1x Pen/Strep (Invitrogen), and 1x GlutaMAX (Invitrogen)) at 37°C for 30-120 minutes depending on sample size. After several washing steps with DMEM/F12+++ medium, the remaining cell pellet was resuspended in GFR Matrigel (Corning) and cultivated in human PDAC organoid medium DMEM/F12+++ supplemented with Wnt3a-conditioned medium (50% *v*/*v*), noggin-conditioned medium (10% *v*/*v*), RSPO1-conditioned medium (10% *v*/*v*), B27 (1x, Invitrogen), nicotinamide (10 mM, Sigma-Aldrich), gastrin (1 nM, Sigma-Aldrich), N-acetyl-L-cysteine (1 mM, Sigma-Aldrich), primocin (1 mg/ml, InvivoGen), recombinant murine epidermal growth factor (mEGF, 50 ng/ml, Invitrogen), recombinant human fibroblast growth factor 10 (hFGF10, 100 ng/ml, PeproTech), A-83-01 (0.5 *μ*M, Tocris Bioscience), and N2 (1x, Invitrogen).

### 2.3. Immunohistochemistry (IHC) Stainings and Imaging

Sections from paraffin-embedded primary PDAC tissue samples were provided by the Tumor- and Normal Tissue Bank of the Institute of Pathology, University Hospital Carl Gustav Carus. The hematoxylin-eosin (H&E) and IHC stainings for KRT81 (Santa Cruz, #sc100929, 1 : 150) and CFTR (Abcam, #ab131553, 1 : 300) were performed according to a standard protocol on deparaffinized tissue sections. Images were taken by an EVOS FL Auto (Life Technologies) microscope. CFTR expression was considered to be positive if a medium to strong staining was detected in more than 10% of the epithelial cells. Analysis of KRT81 stainings was done according to the criteria of Muckenhuber and colleagues [7]. In brief, only a strong staining of KRT81 in at least 30% of epithelial cells leads to the classification of a “KRT81-positive PDAC.” Organoid lines NR002, NR005, and NR006 were derived from fine needle aspirations, so no primary tumor tissue was available to perform IHC stainings.

### 2.4. Immunofluorescence Stainings

Whole PDAC organoids were collected in 15 ml falcons, fixed in 2% formaldehyde (Sigma-Aldrich) over night at 4° C, permeabilized with 0.3% Triton X-100 (Sigma-Aldrich) for 20 minutes, and blocked with 1% BSA (Thermo Fisher Scientific) and 0.1% Triton X-100 in PBS (Sigma-Aldrich) for 1 hour. Samples were then incubated with primary antibodies against KRT81 (Santa Cruz, #sc100929) or CFTR (Abcam, #ab131553), both diluted 1 : 50 in blocking buffer, for 2 hours at room temperature, followed by a 1-hour incubation step with the secondary goat-anti-rabbit Alexa-Fluor 488 antibody (Life Technologies), diluted 1 : 200 in blocking buffer. PDAC organoids additionally were stained with DAPI (Thermo Fisher Scientific) and Alexa-Fluor 568 phalloidin (Thermo Fisher Scientific). Images were taken by Zeiss LSM 510/880 confocal microscope and analyzed with ImageJ (NIH). All acquired images were taken with identical settings.

### 2.5. In Vitro Drug Assays

Mechanically dissociated PDAC organoids were plated in 384 well plates in 15 *μ*l Matrigel supplied with 40 *μ*l PDAC organoid medium. 24 h later, drug treatment was started with conventional chemotherapeutic drugs diluted as follows: gemcitabine (1 *μ*M, 200 nM, 100 nM, 50 nM, 25 nM, 10 nM, and 1 nM), 5-fluorouracil (5-FU; 50 *μ*M, 25 *μ*M, 10 *μ*M, 5 *μ*M, 1 *μ*M, 100 nM, and 10 nM), oxaliplatin (500 *μ*M, 100 *μ*M, 50 *μ*M, 25 *μ*M, 10 *μ*M, 1 *μ*M, and 100 nM), irinotecan (250 *μ*M, 25 *μ*M, 10 *μ*M, 1 *μ*M, 100 nM, 10 nM, and 1 nM). Negative controls and each drug dilution were done in triplicates. Medium was replaced after 72 h. Readout was done after 144 h incubation by measuring the metabolic activity using PrestoBlue cell viability reagent (Invitrogen) following the manufacturer's protocol. Briefly, organoids were incubated for 2 hours at 37°C with PrestoBlue and fluorescence measured at 560/590 nm using a Varioskan LUX (Thermo Scientific). Relative viability was calculated after blank subtraction by normalizing to the mean of the negative control. All drug assays were carried out three times. In order to dissect subtype specific drug responses assay results from the quasimesenchymal (KRT81^+^) and double-positive (KRT81^+^/CFTR^+^) organoid lines were combined. The same was done for the drug assay results from the exocrine-like (CFTR^+^) and classical (KRT81^−^/CFTR^−^) PDAC organoid lines.

### 2.6. Quantitative Real-Time PCR (qPCR)

Total RNA was isolated from organoid cultures using the RNeasy Mini Kit (Qiagen) following the recommended user instructions. cDNA synthesis from 0.5 *μ*g RNA was done with the qScript cDNA SuperMix (Quantabio). qPCR was carried out using the GoTaq qPCR Master Mix (Promega) on a StepOnePlus Real-Time PCR System (Thermo Fisher Scientific) for expression analysis of the following genes: GAPDH (5′-GCA CCA CCA ACT GCT TAG-3′ (sense), 5′-ATG ATG TTC TGG AGA GCC CC-3′ (antisense)); ACTB1 (5′-AAA TCT GGC ACC ACA CCT TC-3′ (sense), 5′-AGA GGC GTA CAG GGA TAG CA-3′ (antisense)); HNF1A (5′-ACG ACG ATG GGG AAG ACT TC-3′ (sense), 5′-GAC TTG ACC ATC TTC GCC AC-3′ (antisense)); and CFTR (5′-CGT CAT CAA AGC ATG CCA AC-3′ (sense), 5′-TCG TTG ACC TCC ACT CAG TG-3′ (antisense)). Calculation of the relative gene expression was done as described by Hellemans and colleagues ([17]). Briefly, arithmetical means were calculated for each gene from all analyzed samples for conversion of quantification cycle values into relative quantities (RQs). Next, the geometrical mean of the RQs from the two housekeeping genes was calculated resulting in the sample-specific normalization factor (NF). The relative expression was determined by dividing the RQ with the NF.

### 2.7. Statistical Analysis

Correlation analysis was done by calculating the Pearson correlation coefficient in GraphPad Prism (version 6.02), assuming a normal distribution of the data. Confidence interval was 95% (two-tailed) for *p* value calculation.

## 3. Results

### 3.1. Generation of a Human Pancreatic Cancer Organoid Biobank

**Figure 1:**
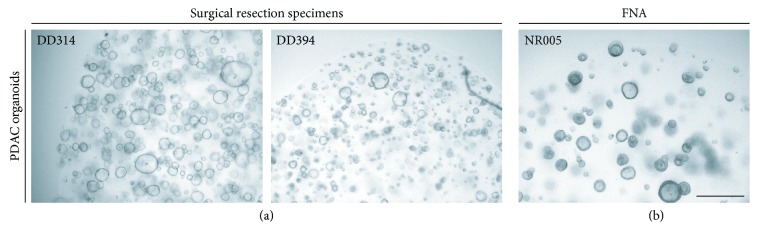
Establishing a human PDAC organoid bank. Phase-contrast images of three representative established PDAC (passage > 10) organoids derived from (a) surgical resection specimens (DD314 and DD394) and (b) EUS-guided fine needle aspiration (NR005). Scale bars represent 500 *μ*M.

We collected human primary tumor samples from 31 PDAC patients that were treatment-naïve: 25 specimens from surgical tumor resections and 6 from endoscopic ultrasound- (EUS-) guided fine needle aspiration (FNA) (Figure 1). The total organoid generation efficiency was 71% yielding 22 PDAC organoid lines (68% for surgical resections and 83% for FNA). All primary cancers were histologically confirmed PDACs. Criteria for new PDAC organoid lines were a stable growth for more than 10 passages and the presence of mutated KRAS, analyzed by Sanger sequencing or—if needed—Illumina panel sequencing, thus excluding growth of normal pancreatic organoids, a common problem in PDAC organoid generation [11]. Specimens from FNAs showed a higher outgrowth efficiency compared to resection specimens. Overall, growth rates are comparable to previously published organoid biobanks [8, 11].

### 3.2. CFTR Might Constitute an Alternative to HNF1A as a Biomarker for PDAC Subtyping

**Figure 2:**
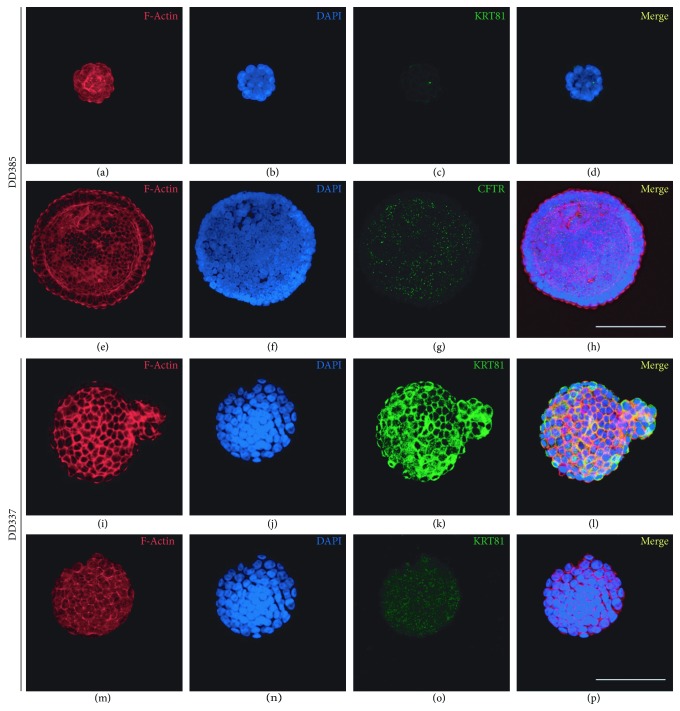
Confocal CFTR and KRT81 immunofluorescence analysis of human pancreatic cancer organoids. Representative stainings of two PDAC organoids (DD385 and DD337) depicting CFTR^+^/KRT81^−^ (a–h) and CFTR^−^/KRT81^+^ (i–p) subtypes, respectively. Scale bars represent 200 *μ*m.

Due to the lack of a suitable HNF1A antibody, we searched for an alternative marker for subtyping PDAC. CFTR is part of the PDAssigner gene set defining the exocrine-like subtype [4]. We therefore performed immunofluorescence (IF) stainings of CFTR as well as KRT81 in 10 organoid lines (Figure 2 and Supplementary Figure S1). The organoid lines DD314, DD376, DD385, DD394, and DD442 were CFTR positive (CFTR^+^), whereas no significant expression of KRT81 (KRT81^−^) was observed. On the other hand, the organoid lines DD337, DD439, and NR006 exhibited no CFTR immunoreactivity (CFTR^−^), but a strong KRT81 positivity (KRT81^+^). No expression of both markers (KRT81^−^/CFTR^−^) was observed for NR005, while NR002 expressed both markers (KRT81^+^/CFTR^+^). Thus, CFTR and KRT81 showed a mutually exclusive expression pattern, assigning nearly all PDAC organoids to the described two most frequently occurring subtypes: exocrine-like (CFTR^+^/KRT81^–^) and quasimesenchymal (CFTR^–^/KRT81^+^). In addition, one double-negative “classical” (KRT81^−^/CFTR^−^) and one double-positive organoids were contained within the analyzed cohort.

**Figure 3:**
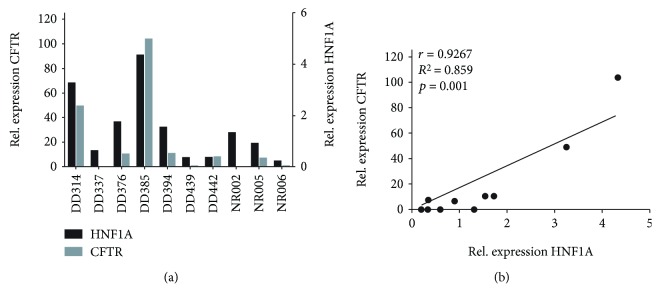
CFTR and HNF1a expression correlate in human PDAC organoids. (a) Relative gene expression of *HNF1A* and *CFTR* in 10 analyzed human PDAC organoid lines. Organoids have been established from surgical resection specimens (DD314-DD442) or EUS-guided FNA samples (NR002-NR006). RT-qPCR data were analyzed by including the two housekeeping genes, GAPDH and ACBT1. (b) Calculation of the Pearson correlation coefficient shows a high linear relationship between the mRNA level of *HNF1A* and *CFTR* (*r* = 0.927) within the analyzed PDAC organoid lines (*p* value = 0.001 (two-tailed), *α* = 0.05).

To further establish CFTR as an alternative marker for HNF1A, we analyzed the mRNA expression levels of both genes in all PDAC organoid lines by qPCR (Figure 3(a)).

Based on a cutoff value for positivity for HNF1A and CFTR of 2-fold overexpression, the organoid lines DD314, DD376, DD385, DD394, DD442, and NR005 were judged positive for both genes, while the organoid lines DD337, DD439, and NR006 were considered to be negative for both genes. The organoid line NR002 was positive for HNF1A, while negative for CFTR. A strong linear correlation between the expressions of HNF1A and CFTR was detected (*r* = 0.927; *p* = 0.001; Figure 3(b)).

Comparing IF and qPCR results, the organoid lines DD314, DD376, DD385, DD394, and DD442 were CFTR positive both on the mRNA and the protein levels, whereas DD337, DD439, and NR006 were negative on both levels. For two cases (NR002, NR005), mRNA levels did not match with the corresponding IF stainings. NR002 showed a weak but clearly present expression of CFTR on protein level, while it was judged negative based on the mRNA level. The opposite was seen for NR005, where mRNA levels of CFTR were judged positive, while no protein expression was detected.

### 3.3. Preservation of Subtypes between Organoid Lines and Their Corresponding Primary Tumors

To answer the question if PDAC organoids express the same subtype-specific immunoreactivity as their corresponding primary tumor, we performed immunohistochemical stainings for KRT81 and CFTR on paraffin sections for all patients of which organoids were derived from resection specimens (*n* = 7; Supplementary Figure S2). CFTR and KRT81 expression was consistent with the organoid immunoreactivity for organoid lines DD314, DD337, DD376, DD385, DD394, and DD442. For DD439, IHC staining was positive for both markers, whereas the corresponding organoid line only expressed KRT81. In summary, in 6/7 samples, the subtype was preserved between primary tumors and organoid line.

### 3.4. Drug Response Testing to Conventional Chemotherapy

**Figure 4:**
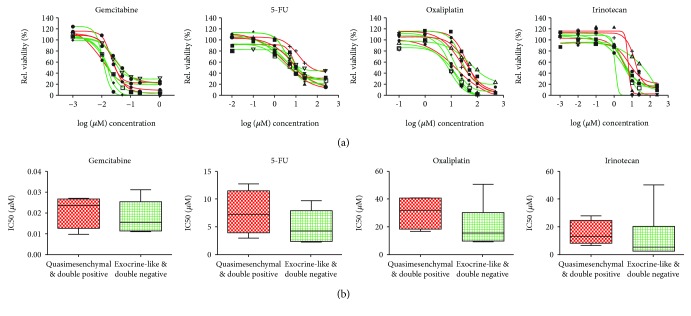
Drug response of PDAC organoid lines. (a) PDAC organoid response to different concentrations of gemcitabine, 5-FU, oxaliplatin, and irinotecan. Red curves indicate KRT81^+^ (quasimesenchymal/double positive) and green curves KRT81^−^ (exocrine-like/classical) PDAC organoid lines. Relative viability was calculated by normalizing to the mean of the negative control. Drug response curves were calculated in GraphPad Prism (version 6.02) via nonlinear regression (curve fit) analysis, requiring drug concentrations to be log transformed. (b) Comparison of IC50 values between all analyzed quasimesenchymal/double-positive and exocrine-like/classical PDAC organoids for gemcitabine, 5-FU, oxaliplatin, and irinotecan. Box plots show the median values and the upper and lower quartile values in each group.

To address whether the different molecular subtypes exhibit a differential drug response towards conventional chemotherapeutics, PDAC organoids were treated with gemcitabine and the single drug compounds of the FOLFIRINOX regimens, irinotecan, oxaliplatin, and 5-FU (Figure 4(a)). A wide variation in drug response was observed for each drug. In order to perform a group comparison, the KRT81^+^ quasimesenchymal/double-positive (*n* = 4) organoid lines and the KRT81^−^ exocrine-like/classical (*n* = 6) organoid lines were combined. A nonsignificant (*p* > 0.05; Figure 4(b)) 1.5-fold higher resistance of KRT81^+^ organoids against 5-FU (mean IC50 KRT81^+^ 5.01 *μ*M; KRT81^−^ 7.52 *μ*M) and oxaliplatin (mean IC50 KRT81^+^ 20.88 *μ*M; KRT81^−^ 30.36 *μ*M) was observed, while no difference was detected for gemcitabine (mean IC50 KRT81^+^ 0.02 *μ*M; KRT81^−^ 0.02 *μ*M) and irinotecan (mean IC50 KRT81^+^ 7.61 *μ*M; KRT81^−^ 6.33 *μ*M).

## 4. Discussion

For many years, classical two-dimensional cell cultures in plastic dishes were the workhorse of cancer research. Based on the identification of Lgr5 as a marker gene of intestinal stem cells [18], a novel three-dimensional culture system named organoids was developed that faithfully recapitulates the tissue of origin [19]. This was possible by using a matrix (Matrigel) plus a defined cocktail of growth factors and inhibitors based on the growth requirements of normal intestinal stem cells [20]. In the meantime, several different organoid culture protocols have been published for many different organs [21]. Following the initial establishment of normal tissue organoid cultures, also organoids derived from human tumors were described [13, 22]. These patient-derived cancer organoids open up new opportunities for personalized therapy, as they mimic *in vitro* to a high-degree response of the tumor *in vivo* [14].

PDAC is a very heterogeneous disease, both on the histological and on the molecular levels [23]. Nevertheless, currently, nearly all patients receive the same chemotherapeutic treatment, as molecular subtyping has not entered the clinical stage yet. There is a tremendous need in defining better treatment strategies, as most patients present with advanced disease stages and systemic chemotherapies in general have only a minor effect on overall survival. Grouping of PDAC into different molecular subtypes has shown to distinguish patients with different survivals [4, 7]. Collisson et al. described three different PDAC subtypes: a classical, a quasimesenchymal, and an exocrine-like subtype. This subtyping was based on mRNA expression analyses using microarrays of laser capture-microdissected material. To facilitate the cumbersome and also decay prone mRNA-based subtyping, Noll et al. identified the protein markers HNF1A and KRT18, which can classify PDAC into the established “Collisson” subgroups [5]. In a follow-up study, the authors further established the value of HNF1A/KRT81-based subtyping by documenting differential therapeutic response of patients to standard of care treatment [7].

As all these studies have been performed on patient material in a retrospective setting, prospective clinical trials have to show that personalizing treatment according to the described molecular subtypes is of benefit to PDAC patients. In order to set up such a clinical trial, two points are of importance: firstly, the subtyping needs to be rather fast, and secondly, very reproducible. Especially in the neoadjuvant setting, only very little tumor material is available, since material is mostly received from EUS-guided FNAs. This material does not suffice on a routine basis to perform IHC-based subtyping. Organoids can be generated with a high efficiency from FNAs (in our current cohort in 83%) and therefore constitute a feasible way to expand tumor material ex vivo to perform subtyping. The second important point is reproducibility of the IHC stainings. The originally used HNF1A antibody (#sc-8986, Santa Cruz Biotechnology) was discontinued and is no longer available. Testing of alternative antibodies for HNF1A has resulted in contradictory and inconclusive results. We therefore set out to establish CFTR as a substitute for HNF1A on PDAC organoid cultures. CFTR IF staining gave either very strong or very weak/absent stainings, allowing us to classify organoid lines as either positive or negative. As we could not perform HNF1A IHC to compare side by side HNF1A to CFTR stainings, we performed mRNA expression analyses. This resulted in a highly significant correlation of the transcript levels of the two genes. In line with previously published papers, CFTR expression was mutually exclusive to KRT81 expression in nearly all organoid lines we analyzed. We could therefore assign—assuming that CFTR can indeed substitute HNF1A—our organoid lines into the quasimesenchymal, the exocrine-like, or classical subtype. In addition, one double-positive organoid line was detected. The existence of PDACs expressing both markers was previously observed [5, 7].

However, the existence of the exocrine-like subtype was recently questioned and attributed to a contamination of normal pancreatic tissue in the analyzed samples [24]. As our cultures contain only tumor cells based on the allele frequency found for the KRAS mutation and the homogenous positivity of the whole cultures in IF stainings, our data nevertheless argues for existence of this PDAC subtype. Noteworthy, we observed a high concordance between the PDAC subtypes of the primary tumor and the respective organoid lines (6/7). In only one case (DD439), a signal for CFTR and KRT81 was seen in immunohistochemical stainings of the primary tumor, whereas the corresponding organoid line only showed a high KRT81 expression on protein and mRNA level. A possible explanation could be a restricted clonality of this PDAC organoid line. A primary tumor was judged CFTR positive if a minimum of 10% of epithelial cells were stained. It is therefore possible that the organoid line was established from a CFTR-negative region, which in this particular case is up to 80% of the primary tumor. In any case, one limitation of the present analysis is the lack of microarray-based mRNA expression subtyping of the organoid lines using the original PDAssigner gene set of Collisson et al. as a control.

Drug assays performed for the frequently used chemotherapeutics in PDAC treatment did not show a statistically significant differential effect between KRT81^+^ (quasimesenchymal/double positive) and KRT81^−^ (exocrine-like/classical) PDAC organoid lines. Collisson et al. have described quasimesenchymal PDAC 2D cell lines to be more sensitive to gemcitabine compared to the classical subtype [4]. Muckenhuber and colleagues suggest KRT81^+^ tumor cells to be more resistant to the FOLFIRINOX regimen compared to the exocrine-like subtype (HNF1A^+^). In line with this, we observed a tendency of KRT81^+^ PDAC organoids to be more resistant towards 5-FU and oxaliplatin in our cohort, comprising 2/3 drugs of the FOLFIRINOX regimen, although we could clearly document the feasibility of drug testing in primary patient-derived tumor models such as the organoid system. Larger PDAC organoid libraries in conjunction with the KRT81/CFTR-based subtyping approach might reveal in the future subtype-specific resistance patterns towards conventional or targeted drugs.

In summary, subtyping of FNA-derived PDAC organoid lines based on CFTR and KRT81 might constitute a feasible way to perform prospective clinical trials for the evaluation of subtype-specific personalized treatment protocols.

## Data Availability

The quantitative real-time PCR data for the expression analysis of HNF1A and CFTR that have been used to support the findings of this study are available from the corresponding author upon request. Images taken by the Zeiss LSM 510/880 confocal microscope from the immunofluorescence stainings of CFTR and KRT81 from organoid lines D314, DD376, DD394, DD439, DD442, NR002, NR005, and NR006 are included within the supplementary information file (Supplementary Figure S1).

## Supplementary Materials

Supplementary 1 Supplementary Figure S1: confocal CFTR and KRT81 immunofluorescence analysis of human PDAC organoids. CFTR and KRT81 immunofluorescent stainings of eight additional PDAC organoid lines. Lines were classified as CFTR^+^/KRT81^−^ (DD314, DD376, DD394, and DD442), CFTR^−^/KRT81^+^ (DD439 and NR006), CFTR^−^/KRT81^−^ (NR005), and CFTR^+^/KRT81^+^ (NR002). Scale bars represent 200 *μ*m.

Supplementary 2 Supplementary Figure S2: CFTR/KRT81 IHC staining of paraffin-embedded primary PDAC sections. Corresponding primary tumor tissue sections of organoids from surgical PDAC specimens were immunohistochemically stained for KRT81 and CFTR. Lines were classified as CFTR^+^/KRT81^−^ (DD314, DD376, DD385, DD394, and DD442), CFTR^−^/KRT81^+^ (DD337), and CFTR^+^/KRT81^+^ (DD439). Scale bars represent 1000 *μ*M and 200 *μ*M.
