# Erratum to “CFTR Expression Analysis for Subtyping of Human Pancreatic Cancer Organoids”

**DOI:** 10.1155/2019/9209764

**Published:** 2019-09-18

**Authors:** Alexander Hennig, Laura Wolf, Beatrix Jahnke, Heike Polster, Therese Seidlitz, Kristin Werner, Daniela E. Aust, Jochen Hampe, Marius Distler, Jürgen Weitz, Daniel E. Stange, Thilo Welsch

**Affiliations:** ^1^Department of Visceral, Thoracic and Vascular Surgery, Medical Faculty and University Hospital Carl Gustav Carus, Technische Universität Dresden, Dresden, Germany; ^2^National Center for Tumor Diseases (NCT), Dresden, German Cancer Research Center (DKFZ), Heidelberg, Faculty of Medicine and University Hospital Carl Gustav Carus, Technische Universität Dresden, Dresden, Helmholtz-Zentrum Dresden-Rossendorf (HZDR), Dresden, Germany; ^3^Institute of Pathology and Tumour- and Normal Tissue Bank of the University Cancer Center (UCC), University Hospital Carl Gustav Carus, Medical Faculty, Technische Universität Dresden, Dresden, Germany; ^4^Medical Department I, Medical Faculty and University Hospital Carl Gustav Carus, Technische Universität Dresden, Dresden, Germany

In the article titled “CFTR Expression Analysis for Subtyping of Human Pancreatic Cancer Organoids” [1], there was an error in Figure 2, where panel (o) should be labelled as “CFTR” not “KRT81.” This occurred due to a production error. The corrected figure is shown below.

## Figures and Tables

**Figure 1:**
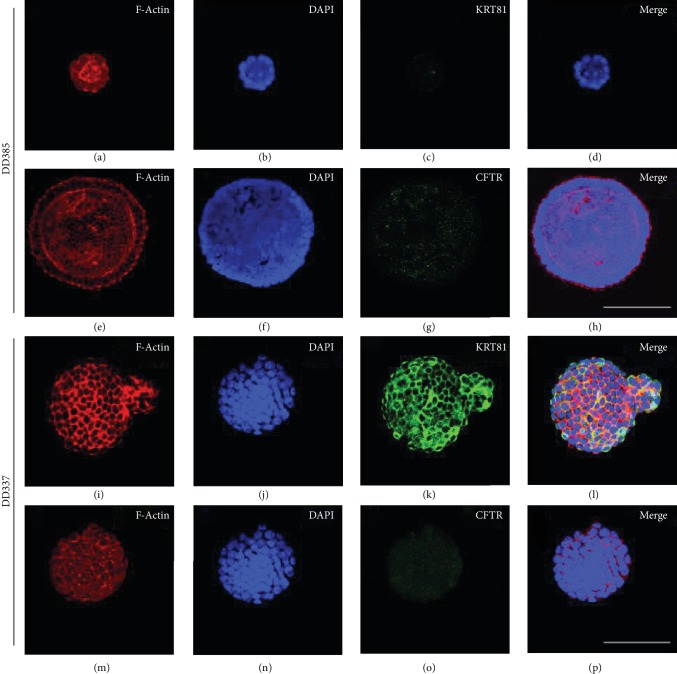
Confocal CFTR and KRT81 immunofluorescence analysis of human pancreatic cancer organoids. Representative stainings of two PDAC organoids (DD385 and DD337) depicting CFTR^+^/KRT81^−^ (a–h) and CFTR^−^/KRT81^+^ (i–p) subtypes, respectively. Scale bars represent 200 *μ*m.
